# Mobility Problems and Weight Regain by Misdiagnosed Lipoedema After Bariatric Surgery: Illustrating the Medical and Legal Aspects

**DOI:** 10.7759/cureus.5388

**Published:** 2019-08-14

**Authors:** Sjaak Pouwels, Hendrika J Smelt, Mohammed Said, Johannes F Smulders, Maarten M Hoogbergen

**Affiliations:** 1 Surgery, Haaglanden Medisch Centrum, The Hague, NLD; 2 Surgery, Catharina Ziekenhuis, Eindhoven, NLD; 3 Plastic Surgery, Catharina Ziekenhuis, Eindhoven, NLD

**Keywords:** lipoedema, arthritis, bariatric surgery, plastic surgeon, medical and legal aspects, insurance

## Abstract

Lipoedema is a progressive disorder that is characterized by an abnormal distribution of subcutaneous adipose tissue, which results in a disproportion between the extremities and the trunk. This vascular/dermatological disease might have a detrimental impact on psychosocial wellbeing and quality of life. In this article, we report on a patient with morbid obesity that had a Roux en-Y Gastric bypass with sufficient weight loss. However, due to this weight loss, an abnormal disproportion came to light. A dermatologist diagnosed lipoedema five years after the surgery. Eventually, she had a dermolipectomy of the upper arms, of which reimbursement was initially rejected by her insurance.

## Introduction

Lipoedema is a chronic and often progressive disorder, which is characterized by an abnormal distribution of subcutaneous adipose tissue [1]. This often results in a pronounced disproportion between the extremities and the trunk. The etiology of this disease is not fully understood, but it is thought to be multifactorial [1-2]. One of the possible explanations is a symmetrical increase in subcutaneous adipose tissue in the lower and/or upper extremities [3]. Other findings include the accumulation of fluid (e.g., orthostatic edema) that results in pain, tenderness, and sensitivity to pressure. Together with easy bruising, these are the sequelae that patients with lipoedema often describe [1-3]. A chronic and progressive disorder like lipoedema is frequently associated with impairment in psychosocial wellbeing and quality of life [4]. This disorder affects mostly women, and the onset of lipoedema is usually in periods of hormonal changes like puberty, pregnancy, or menopause [1]. This also might be attributed to the autosomal dominant inheritance pattern with sex limitation [2]. Unfortunately, only parts of the pathophysiology of lipoedema are understood and the specific roles of hormones and genetics still need to be clarified [1-3,5].

Lipoedema can be divided into three morphological stages, of which the first stage is characterized by a homogenous increase in subcutaneous fatty tissue and the skin is still smooth. The second stage of lipoedema is associated with an irregular skin surface and shows nodular changes in the subcutaneous tissue. The final and third stage indicates a pronounced increase in circumference with very loose skin, also called a ‘dewlap’ [1-2]. Unfortunately, with this disease classification, disease progression is not predictable and, therefore, may vary on a case-to-case basis [1]. In case of comorbidities, of which inactivity and obesity are often present, progression can occur due to the secondary development of lymphedema. This can occur in any stage of the disease and, of course, can aggravate clinical symptoms [1-2,5].

In patients scheduled for bariatric surgery, diagnosing lipoedema can be challenging and, therefore, can be easily confused with obesity. The diagnosis is frequently unrecognized and is often misdiagnosed [2,6]. It is very important that in case of clinical suspicion, a dermatologist is consulted in an early stage and preferably prior to bariatric surgery. This is because even after bariatric surgery, lipoedema is progressive, results in gradual enlargement of fatty deposition, and causes impaired mobility and further comorbidities like osteoarthritis and lymphatic insufficiency [3-4,7] The earlier mentioned disproportion becomes more pronounced when obesity and lipoedema coexist and patients lose a significant amount of weight after bariatric surgery [7]. This cannot be treated with dieting or physical exercise and often results in considerable frustration and psychological issues [2,7]. Treatment options for lipoedema consist of complex decongestive therapy and surgery by microcannula tumescent liposuction. Liposuction is the only available treatment capable of reducing the pathological adipose tissue durable and to prevent complications [3,5]. In this report, we present a patient who has been diagnosed with stage three lipoedema approximately five years after bariatric surgery.

## Case presentation

A 63-year-old woman, with a medical history of arthritis and a transient ischemic attack, had a length of 168 cm and weight of 200 kg, resulting in a body mass index (BMI) of 70 kg/m², presented to the hospital. In connection with arthrosis, she needed to lose weight and, therefore, she achieved a weight loss of 43 kg to 157 kg (BMI 57.7 kg/m²) with a conservative diet effort and physical exercise. Sports were not possible due to arthritis and the abnormal distribution of adipose tissue in the upper arms and legs up to the ankles. She could have been eligible for knee prosthesis of both her knees if she lost more weight. However, her weight stabilized at 157 kg. Therefore, her orthopedic surgeon referred her to the bariatric surgery outpatient clinic and she underwent a laparoscopic Roux-en Y Gastric Bypass in 2012. Her weight was 85 kg (BMI 30.1 kg/m²) two years after surgery. At this stage, a significant and considerable change was found in her quality of life. However, after a 72-kg weight loss, she had a lot of excess skin (upper arms and legs). The excess skin disturbed physical exercise enormously. Besides that, the circumference of her legs was not much reduced.

**Figure 1:**
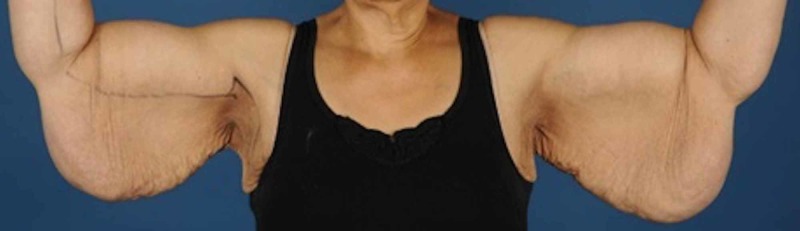
Upper arms before dermolipectomy

**Figure 2:**
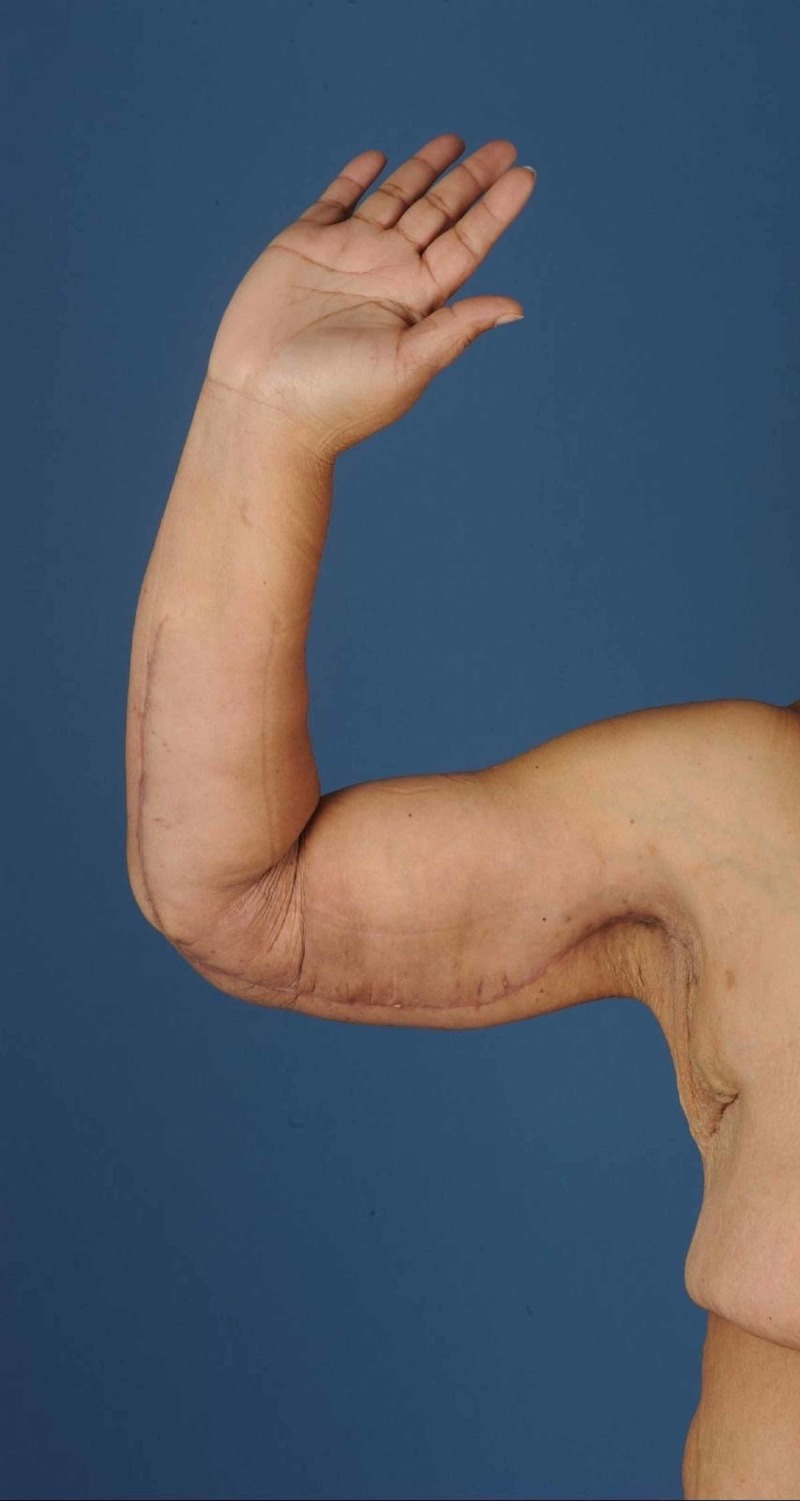
Right upper arm after dermolipectomy

**Figure 3:**
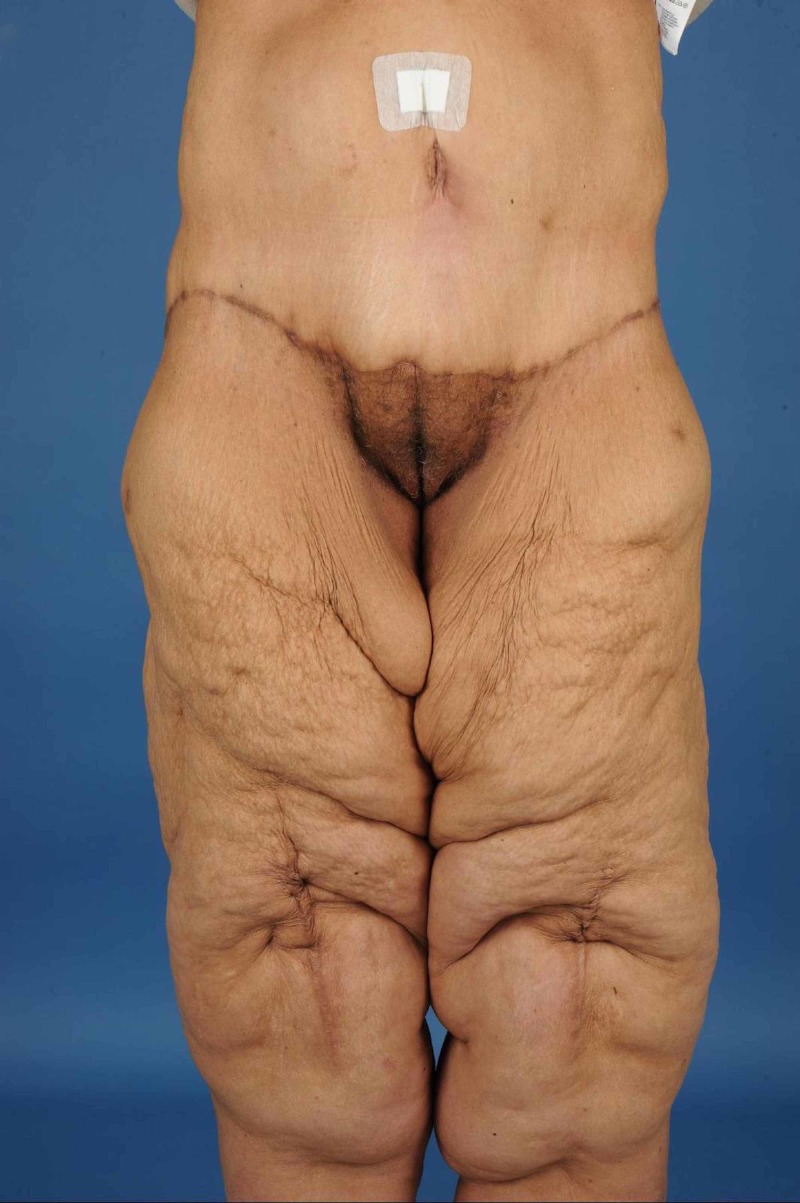
Legs after 115 kg of weight loss

**Figure 4:**
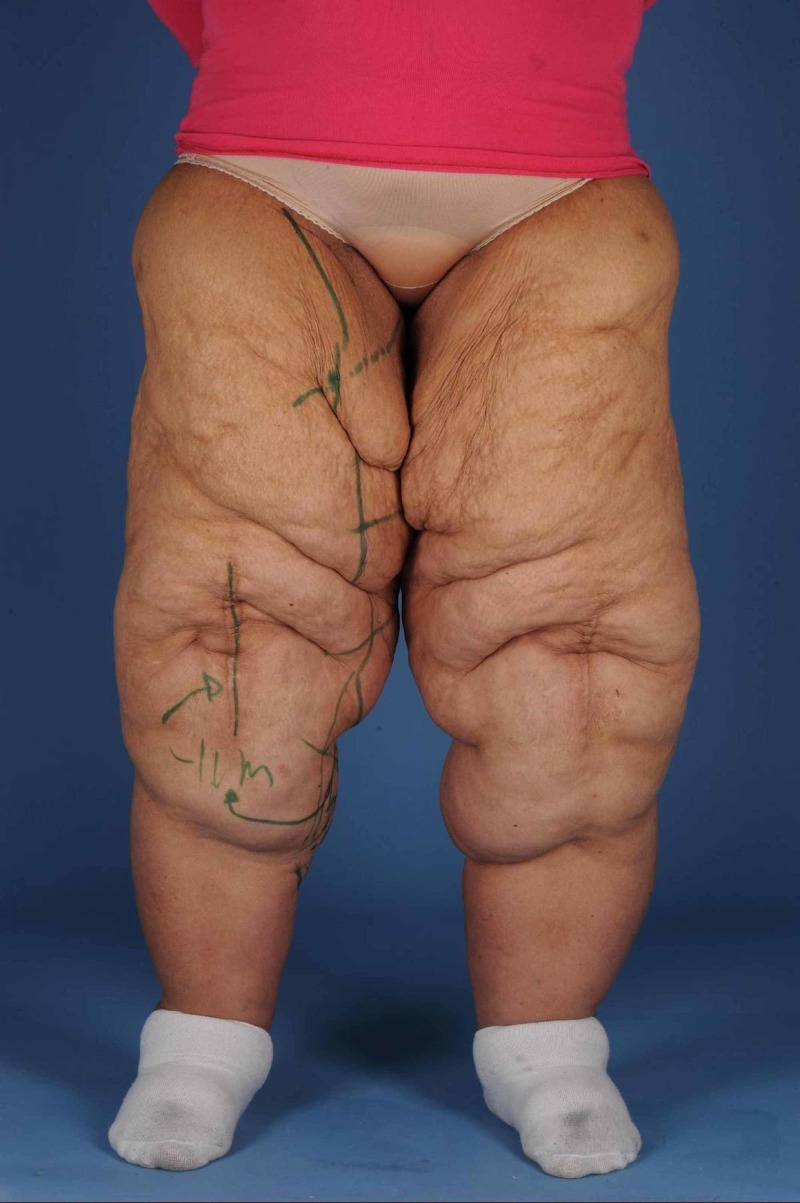
Legs after 30 kg of weight regain

In 2014, she underwent a lower body lift and a dermolipectomy of the upper arms in 2015 (Figures 1-2). Dermolipectomy of the legs was rejected by her insurance. In 2016, she regained weight till 115 kg (BMI 40.7 kg/m²), which especially resulted in a large increase in the circumference of her upper legs (Figures 3-4). In addition, her mobility reduced. The situation seemed suspicious of lipoedema and, therefore, she was referred to the dermatologist. The dermatologist diagnosed lipoedema stage III, for which no good conservative treatment option was available. The plastic surgeon suggested liposuction and skin removal for this deformity. 

## Discussion

As mentioned earlier, and as also the case in our patient, lipoedema is a progressive and chronic disease that has a significant negative impact on self-esteem, mobility, and quality of life. In advanced stages of the disease, there is mainly bulging localized often on the inner sides of the thighs and knees and rarely around the ankles [1-7]. Rubbing and friction cause micro tissue trauma and occlusion effects in the skin folds, which can cause maceration and eventually will result in infections [1-4]. Along with the significant morbidity caused by advanced stages of lipoedema, it also induces biomechanical changes. The earlier-mentioned bulging on the inner aspects of the thighs results in an impaired walking pattern, due to alterations in the mechanical axis of the legs [1,3-4]. Eventually, this can result in significant orthopedic complications like primarily valgus osteoarthritis of the knee [1].

When correctly diagnosed, patients will have expectations of their treatment, so as a physician, it is important that the expectations regarding the restoration or rehabilitation of body morphology be clearly managed [6]. This is the case in patients after bariatric surgery but can be even more challenging in patients with obesity and lipoedema who have very high expectations [7]. Excess skin or disproportions in their body can be unpleasantly surprising and can cause psychological morbidity. Therefore, if necessary, a consultation at the psychology department might be advised [6-7]. This psychological distress can lead to patients' assumptions that they are living in a skin envelope too large for their body, which can cause difficulties in dressing and might even be a cause of embarrassment while being seen in public. It is of the utmost importance to address these psychological sequelae because it can blind patients to their success of the bariatric (surgical) treatment [7]. Sadly enough, it is not uncommon that patients address complaints with a feeling that their treatment was not a success. Finally, patients in need of body-contouring surgery after bariatric surgery often need the support of the hospital due to reimbursement problems with their insurance. Despite these insurance problems, those who have had body-contouring surgery following bariatric surgery are among the most grateful patients in our day-to-day practice of plastic surgery [6]. Unfortunately, insurance companies have a tendency to hinder this second part of the treatment [6]. Dermolipectomies of the arms and thighs require preapproval; it is up to the medical council to confirm or deny functional discomfort or disability [6].

Medicolegal aspects

In this case report, several medical and legal aspects were very important. First, the combination of lipoedema and obesity, especially in bariatric surgical practice, is very rare [3,7]. In general, informed consent is extremely important. Of course, it is the doctor’s obligation to explain the patient's medical condition and the benefits and downsides of every treatment [8]. It is basically a double-edged sword; it can protect both the patients' and doctors' interests, but when not given correctly, informed consent can be used as a litigation strategy. And if this is the case, the burden of proof always falls on the doctor [8-9]. In other words, in case of a lawsuit, doctors must prove that adequate explanation was given and that valid consent was obtained. The second aspect that needs to be elucidated is the patients' expectations. Especially in the fields of bariatric and plastic surgery, it is very important to address what patients can expect from a surgical procedure. Managing expectations is generally done while explaining the pros and cons of treatment [8-9].

In the case of lipoedema and obesity, if known prior to bariatric surgery, it needs to be explained that there might be less weight loss to be expected than compared with patients with only obesity [3,7]. In some cases, there will be weight regain, which will not respond to revisional bariatric surgery [7]. In terms of body contouring surgery after bariatric surgery, other problems will arise. Currently, in the Netherlands, insurance companies cover the expenses for a bariatric surgical procedure, however, in case of excess skin after weight loss after bariatric surgery, insurance companies do not (fully) reimburse expenses for body contouring surgery [10]. This is despite the fact that there is a known relationship between excessive skin, body image dissatisfaction, and quality of life. Patients with excessive skin after bariatric surgery have an increased risk of developing depression and have a lower quality of life [10].

Even when surgeons obtain informed consent and manage expectations correctly, there is still a debate on legal liability after surgery. In a study by Paik et al. [9], legal litigation following body contouring surgery was examined. This study showed that the most common injuries after surgery were disfigurement and the necessitation of a revisional procedure, and the most common cause of action was negligence (more than 80%) [9]. In the case of an iatrogenic injury or disfigurement, there was a significantly higher probability of getting a settlement [9]. Severe iatrogenic injury is understandable, however, in the specific patient population that has both obesity and lipoedema, where disfigurement is the problem. Plastic and bariatric surgeons need to give a lot of attention to managing expectations because some disfigurement (due to the disease) will stay. Of course, we are now speaking of a rare population, and laws might differ per country and/or state [11-12], but informed consent and management of expectations is pivotal in treating patients with lipoedema and obesity.

## Conclusions

Lipoedema is a very difficult diagnosis in patients with obesity scheduled for bariatric surgery. It is important to pay attention to patients with an abnormal distribution of subcutaneous fat in the lower and/or upper extremities before and after bariatric surgery because obesity can mask the presence of lipoedema. The identification of lipoedema before weight-loss surgery may help guide the patient’s expectations after weight loss.

## References

[1] Reich-Schupke S, Schmeller W, Brauer WJ (2017). S1 guidelines: lipedema. J Dtsch Dermatol Ges.

[2] Schmeller W, Hueppe M, Meier-Vollrath I (2012). Tumescent liposuction in lipoedema yields good long-term results. Br J Dermatol.

[3] Wollina U (2017). Lipedema: up-to-date of a long forgotten disease [Article in German]. Wien Med Wochenschr.

[4] Forner-Cordero I, Szolnoky G, Forner-Cordero A, Kemeny L (2012). Lipedema: an overview of its clinical manifestations, diagnosis and treatment of the disproportional fatty deposition syndrome - systematic review. Clin Obes.

[5] Wagner S (2011). Lymphedema and lipedema - an overview of conservative treatment. Vasa.

[6] Capon A (2010). Body reshaping surgery after massive weight loss. J Visc Surg.

[7] Pouwels S, Huisman S, Smelt HJM, Said M, Smulders JF (2018). Lipoedema in patients after bariatric surgery: report of two cases and review of literature. Clin Obes.

[8] Park BY, Kwon J, Kang SR, Hong SE (2016). Informed consent as a litigation strategy in the field of aesthetic surgery: an analysis based on court precedents. Arch Plast Surg.

[9] Paik AM, Mady LJ, Sood A, Lee ES (2014). Beyond the operating room: a look at legal liability in body contouring procedures. Aesthet Surg J.

[10] Monpellier VM, Antoniou EE, Mulkens S, Janssen IMC, van der Molen ABM, Jansen ATM (2018). Body image dissatisfaction and depression in postbariatric patients is associated with less weight loss and a desire for body contouring surgery. Surg Obes Relat Dis.

[11] Ranum D (2014). Commentary on: beyond the operating room: a look at liability in body contouring procedures. Aesthet Surg J.

[12] Ballard WL, Feagle GR (2014). Commentary on: beyond the operating room: a look at legal liability in body contouring procedures. Aesthet Surg J.

